# Insights into psychosocial problems and associated factors among higher education students in Ethiopia: a cross-sectional study

**DOI:** 10.1186/s12889-024-20262-w

**Published:** 2024-10-11

**Authors:** Hailay Tesfay Gebremariam, Million Desalegn Tassew, Frehiwot Sahle Woldemaryam

**Affiliations:** 1 Department of Ethiopian Languages and Literature (Amharic), College of Social Science and Humanities, Arba Minch University, Arba Minch, Ethiopia; 2 Department of Psychology, College of Education and Behavioral Studies, Wolaita Sodo University, Wolaita Sodo, Ethiopia

**Keywords:** Adolescent students, Associated factors, Cross-sectional study, Higher education, Psychosocial problems

## Abstract

While there has been extensive research on well-known psychosocial problems like depression, anxiety, and stress among higher education students, emerging issues such as emotional problems, antisocial behavior, trauma experiences, and academic difficulties are not as thoroughly studied, particularly in the context of Ethiopian higher education students. These updated psychosocial problems are crucial to explore due to their potentially significant impact on students’ academic performance, personal development, and future prospects. Therefore, this study aims to explore the current psychosocial issues faced by adolescent students at Arba Minch University and identify the factors associated with them. To accomplish this objective, a survey questionnaire was distributed to a sample of 300 university students through a cross-sectional study. The survey questionnaire was designed to provide a thorough understanding of the various types of psychosocial problems experienced by the students. The findings revealed that the most prevalent psychosocial problems among higher education adolescent students were emotional problems (6.7% high/severe, 46.3% moderate), antisocial behavior (5% high/severe, 54.7% moderate), trauma experiences (7% high/severe, 23% moderate), and academic problems (8.3% high/severe, 23% moderate). The prevalence of no/low psychosocial problems was 47%, 40.3%, 69.3%, and 68.7%, respectively. Additionally, statistically significant (*p* < 0.05) associated factors to these psychosocial problems were identified: gender for academic problems, religion affiliation for antisocial behavior, trauma experiences, marital status for trauma experiences, living situation during holidays for emotional problems, age for emotional problems and antisocial behavior, and educational sponsorship for antisocial behavior. The study found that students who lacked a support system, such as family or friends, were more likely to experience psychosocial problems. In conclusion, psychosocial problems among adolescent students in higher education are a pressing issue that requires immediate attention. By understanding the challenges faced by these students, universities can implement effective interventions to support their mental well-being.

## Introduction

Psychosocial problems refer to the difficulties that adolescent students in higher education face in various areas related to their social and personal functioning [1]. Adolescents range in age from 10 to 19 years old and account for 24.19% of the global population. It is the second most rapidly growing and changing period of life, after infancy [2–4]. To maintain continuity and stability, individuals, groups, and circumstances must be able to adjust to the rapid changes of life. Many adolescent students in higher education may struggle to manage the psychosocial issues that arise, such as anxiety, failure, and dropping out [5]. According to Timalsina et al. [3], while many adolescents are vulnerable to a variety of psychosocial problems due to the physical and physiological changes that occur during this stage of development [6], these changes are often neglected.

The prevalence of psychosocial problems among adolescents, particularly college-aged students, varies greatly among study settings [6, 7], and they require assistance in developing their self-esteem and effectively dealing with psychological and internal issues. With this assistance, they will be able to excel academically, achieve their goals, stand out from their peers, and successfully adapt to various life situations [2, 5]. According to Bhosale et al. [8], this stage is crucial as it signifies the transition from childhood to adulthood. Psychosocial issues, including behavioral, emotional, and academic difficulties, are highly prevalent in children and adolescents, even in higher education [3]. If these adolescents experience physical injury, psychological trauma, or significant environmental changes, they are particularly vulnerable to psychosocial dysfunction, especially without a strong support system [1]. The adolescent years are pivotal for the development of good mental health. Adolescents with sound mental health lead fulfilling lives, exhibit no signs of psychopathology, and make valuable contributions to their families, communities, and educational institutions [8], provided they receive help through various preventive measures [9].

The overall development of college-aged students is now at risk due to psychosocial problems [10, 11]. A study conducted in the United States (US) found that 55% of students experience depression [12]. In the Indian context, it is assumed that 14–40% of adolescent students have mental health or psychosocial problems [13–15]. The lack of attention to the personal well-being of adolescents, during a key phase of socialization, may lead to psychosocial problems that can persist throughout their lives and reduce society’s socioeconomic productivity [16, 17]. Specifically, it can be argued that proper psychosocial development of adolescents is reflected in sound academic performance, physical health, and adequate social, emotional, and psychological well-being. This ultimately contributes to reducing the risk of psychosocial and behavioral problems, violence, crime, teenage pregnancy, and substance abuse [3, 17]. Similarly, studies conducted globally show that 20% of adolescents encounter at least one behavioral problem [11, 18] and in Lithuania [19] revealed that 54.5% of students were experiencing such psychosocial problems. Grayson [20] also noted that university students face different concerns like disturbances in mood, relationship problems, and destructive behavior which impair their self-concept. Especially in Pakistan, psychosocial problems among university students have received little attention and research.

However, there is still confusion in defining psychosocial problems despite previous research efforts [16, 18]. In their study, Latiff et al. [18] concluded that psychosocial problems refer to emotional and behavioral disorders, which can be categorized as internalizing and externalizing conditions. Half of all lifetime mental disorders begin before the age of 14, and 75% begin by the age of 24 [21–23]. Internalizing disorders include depression and anxiety, while externalizing disorders include delinquency, aggression, educational difficulties, and truancy. Adolescents are mainly affected by their home and school environments [24]. Schools play a vital role in the development of an adolescent, as they spend much time attending school, engaging in extracurricular activities, and completing academic work at home. Schools represent institutions that contribute to the overall educational and socialization process, critical in the personality development of an adolescent [25, 26].

Research suggests that many adolescent students in higher education may not perform below expectations due to a lack of intelligence or cognitive limitations [7]. The psychosocial success of these students can be seen as an indicator of a society’s level of development [27]. These students are influenced by various factors, including developmental, environmental, and educational factors, which are significant societal shifts that take time to manifest. In order for society to flourish amidst rapid change, high levels of psychosocial success are necessary. Achieving this success enables individuals to effectively navigate life’s challenges and avoid trouble [26, 28]. Conversely, a lack of success may be attributed to psychosocial issues that hinder their ability to empathize and engage with others. Academic pursuits that are not tailored or suitable for scholarly coursework will ultimately fail [25, 29].

Therefore, such inconclusive results across various studies highlight the need for continued research and exploration of the phenomenon by researchers. Ongoing studies are essential to deepen our understanding of the complexities involved in the relationship between psychosocial problems and associated factors. Basically, adolescent students usually have some sort of psychosocial and emotional problems [30], but these problems are less studied and later on, they are responsible for developing severe psychopathological conditions. Thus, it is important to study this particular area to control the long-term consequences. Thus, the objectives of this study are: (1) to identify the psychosocial problems faced by adolescent students in higher education, and (2) to explore the associated factors with relation to the psychosocial problems of university students at Arba Minch University. Specifically, it is intended to answer the following research questions:

1. What is the prevalence of psychosocial problems (emotional problems, anti-social behavior, trauma experiences, and academic difficulties) among adolescent students in Ethiopian universities?
2. Is there any association between socio-demographic information of respondents and psychosocial problems (emotional problems, anti-social behavior, trauma experiences, and academic difficulties) among adolescent students in Ethiopian universities?

The findings of this study are expected to positively influence the design of preventive offices and programs, with a primary focus on psychological interventions to enhance the psychosocial health of higher education adolescent students during their adolescence. Understanding the underlying mechanisms that contribute to psychosocial problems is crucial for developing effective interventions and support systems. Additionally, detecting psychosocial dysfunction in early adolescence can be beneficial for providing quality interventions through life skills training and counseling mechanisms for individuals throughout their lives.

## Literature review

### Concepts on psychosocial problems

Psychosocial problems refer to the challenges individuals face in their psychological and social well-being [27, 31]. These problems can arise from various factors, including family issues, financial difficulties, or mental health disorders [32] According to Sharma et al. [24] psychosocial problems are influenced by a combination of individual and environmental factors. At the individual level, factors such as personality traits, coping mechanisms, and cognitive processes play a significant role. For instance, individuals with high levels of neuroticism may be more prone to experiencing psychosocial problems due to their tendency to perceive situations as threatening or stressful [13, 23, 32]. Similarly, individuals with poor coping skills may struggle to effectively manage stressors, leading to the development of psychosocial problems [16]. Environmental factors also contribute to the development of psychosocial problems. Family dynamics, social support networks, and socio-economic status can all impact an individual’s well-being [33]. For example, individuals from dysfunctional families may be more likely to experience psychosocial problems due to a lack of emotional support or exposure to conflict. Similarly, individuals from lower socio-economic backgrounds may face additional stressors related to financial instability [17, 34], which can contribute to the development of psychosocial problems [8, 35]. On the other hand, individuals with strong social support networks may be better equipped to cope with stressors, reducing the likelihood of developing psychosocial problems [21, 25, 36].

Sharma et al. [24] conducted a study in Dehradun and found that 40.5% of people had psychosocial problems. Similarly, other cross-sectional studies conducted in Dehradun and Nepal reported prevalence rates of 31.2% and 17.03%, respectively [15, 25]. In a recent study by Timalsina et al. [3], it was discovered that 12.9% of teenagers in Nepal experienced psychosocial problems. These problems were further categorized as externalizing problems (4.2%), internalizing problems (44.6%), and attention deficit hyperactivity disorder (25.8%). These research findings highlight the unique and significant needs of adolescents, who are the future of the nation. Similarly, Latiff et al. [18] studied the prevalence of psychosocial problems in Malaysia and revealed that the prevalence of psychosocial problems, including externalizing and internalizing, was high. Respondents showed 24.9% at the abnormal level and 20.7% at the borderline level. In addition, 46% had depression, while 59% and 38% had anxiety and stress, respectively. In developing countries like Ethiopia, the scenario of mental health and its interventions is worse compared to developed countries. There are also related studies on psychosocial problems in the Ethiopian context [6, 37], but the available data from school-based or health center settings do not accurately represent the situation. This highlights a lack of serious effort towards addressing the mental health of adolescent students in Ethiopia.

Nonetheless, the concept of psychosocial issues associated with adolescence revolves around experimentation and discovery, requiring adaptation to physical development, redefining roles within families and among peers in school, and the emergence of a more independent way of life [13, 17, 32]. Adolescents often experience more emotional and psychosocial challenges, such as antisocial behavior, traumatic experiences, and academic difficulties, compared to adults [4, 18]. Significant psychological distress can stem from issues related to identity formation and differentiation [33]. The assessment of mental health among university students is inadequate due to a lack of studies on the impact of psychosocial problems on the development of psychopathology, such as depression and anxiety [19, 31]. As a result, students who struggle with psychosocial difficulties and psychopathology may face significant challenges in their daily lives [31, 33]. This can lead to poorer mental health and well-being, a reduced sense of social cohesion, and an increased risk of future instability and conflict [12]. Some studies indicate that at least one in five children and teenagers are affected by a mental health condition, while six million people worldwide, or at least one in ten, suffer from a major emotional disorder [4, 35].

### Associated factors

Numerous interconnected elements can either worsen or alleviate the psychosocial issues that impact students in higher education [4]. These elements can be broadly categorized into four groups: environmental, academic, social, and individual. Each category consists of various components that play a role in influencing students’ mental health and overall well-being, thus affecting their ability to cope with the demands of college life [23, 32]. Many interconnected elements can affect students’ psychosocial issues, particularly in higher education. These elements can be roughly divided into four categories: academic, social, individual, and environmental. Each of these areas contributes to either the development or improvement of the psychological challenges that students encounter [5]. All of these aspects highlight the intricate interactions among different factors that impact students’ psychosocial issues in higher education. By recognizing and addressing these associated factors, institutions can better assist students in managing their mental health, ultimately enhancing their academic performance and overall well-being [18, 21].

During the complex developmental phase of transitioning from childhood to adulthood, adolescents face various challenges [16, 18]. New college students, who are adolescents exposed to independent lifestyles, are likely to experience psychosocial problems such as antisocial behavior, trauma, emotional issues, and academic struggles [31]. This may be due to the newfound freedom from parental and family restrictions that they enjoy in higher education settings. It is important to note that many university students end up in higher education institutions as a last resort, as they may not have chosen a career path due to poor remuneration. The changes in biological, physical, psychosocial maturity, and cognitive capacity of adolescents vary in their ability to cope with the rapid changes [14]. Throughout their university experience, adolescents often suffer from psychosocial problems, including emotional issues like anxiety, depression, and stress, as well as behavioral problems such as educational challenges, conduct disorders, hyperactivity, and substance abuse [36, 38, 39]. Since psychosocial problems are not always easily detected by parents and teachers, they can be overlooked by society, despite being caused by emotional, traumatic, antisocial, and academic factors. In Malaysia, around 10–20% of the 5.47 million adolescents are affected by psychosocial problems [4, 28]. Additionally, severe anxiety, depression, and stress are prevalent among Malaysian secondary school students, ranging from 9 to 11% [2]. Globally, approximately 26% of adolescents aged 16–24 years old have a mental disorder [15].

### Theoretical model of psychosocial problems

**Fig. 1:**
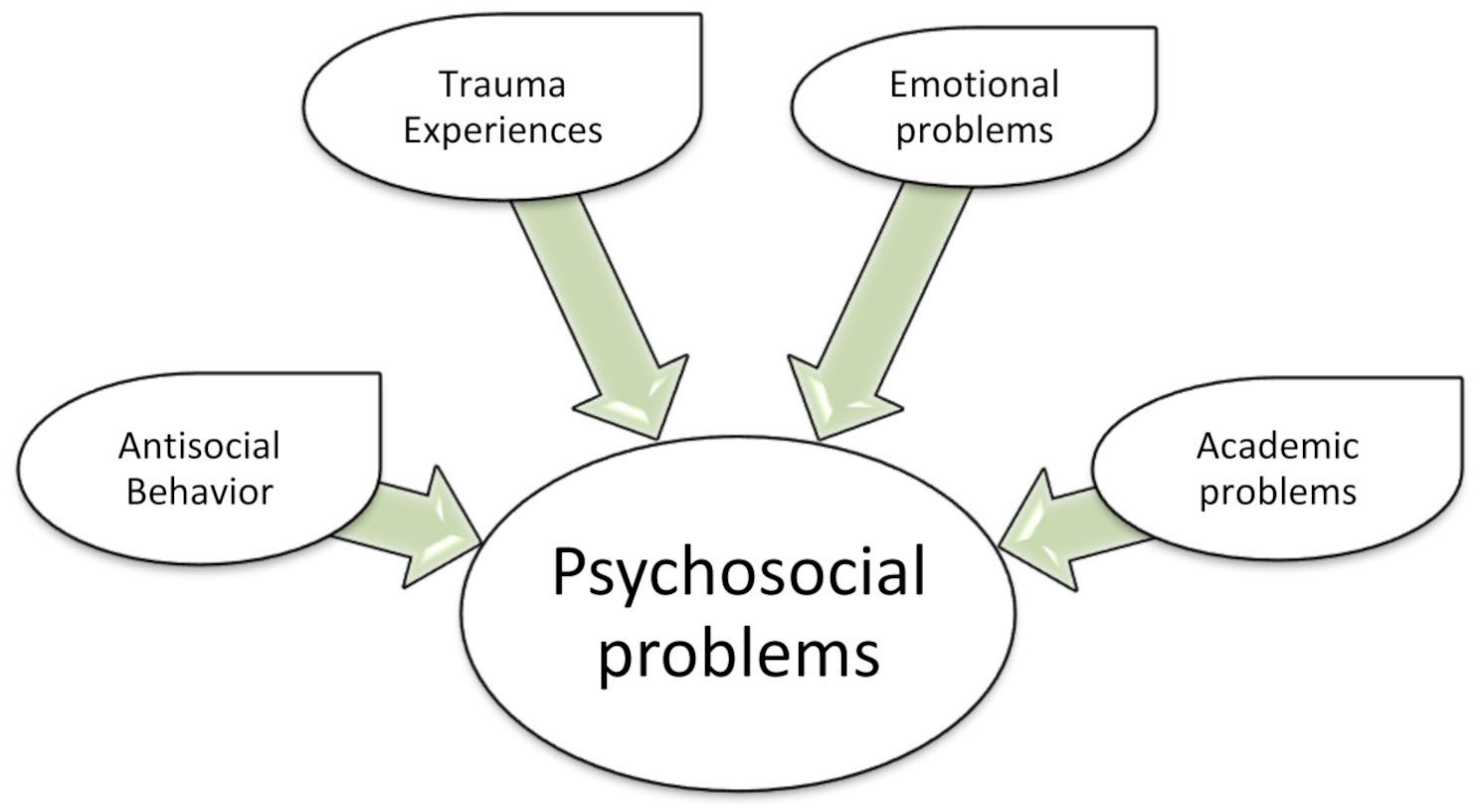
The adapted structural model of psychosocial problems [31]

A theoretical model for the current study was adapted from Nsereko et al. [31], which posited psychosocial problems among African university students in Uganda. The model included (a) individual demographic factors and experiences (such as age, gender, personality characteristics, family issues, student status, personal exposure to traumatic experiences, and socioeconomic status) and (b) contextual factors (such as the sociopolitical environment, the university atmosphere, and peer-related factors; [1, 9, 10, 26]. A list of identified items representing a given factor from the four core factors of psychosocial problems (emotional, antisocial, trauma, and academic) in university students might be applied [31]. Therefore, although there is no validated model in the context of Ethiopian universities and related institutions, the study hypothesized that observed manifest behaviors in individual and environmental experiences influenced the psychosocial status of university students in Ethiopia. The model assumed that academic performance, antisocial behavior, traumatic experiences, and emotional problems had a direct effect on the presence of psychosocial problems (see Fig. 1) among university students.

### The current study

Although previous studies have provided insight into psychosocial issues such as depression, anxiety, and stress that affect higher education students in Ethiopia, they have not fully captured the diversity of these problems. Research by Abera et al. [6] and Wada et al. [7] suggests that various psychosocial, educational, and psychological characteristics may be linked to these issues, significantly impacting academic performance and future well-being. While many studies have focused on depression, anxiety, and stress, it is important to also explore other psychosocial problems among higher education students, especially in Ethiopia. This research is likely to be innovative as it considers psychological problems in conjunction with various factors among university students in Ethiopia. The novelty of this research lies not only in the idea itself but also in the new insights it provides into psychosocial problems such as antisocial behavior, trauma experiences, emotional issues, and academic challenges. Previous studies have primarily concentrated on depression, anxiety, and stress, while others have looked at interventions for these issues. In contrast, this study aims to offer updated insights into psychosocial problems within the context of higher education institutions in Ethiopia. Currently, there is a lack of data on emotional, antisocial, traumatic, and academic problems among higher education students in Ethiopia, particularly at Arba Minch University. Therefore, this study seeks to determine the prevalence of these psychosocial problems and identify the associated factors among higher education students at Arba Minch University in Ethiopia.

## Methodology

### Design and participants

Using a cross-sectional research approach, this study examined the prevalence of psychosocial issues and associated factors among Ethiopian students at Arba Minch University. The research was conducted at a public university in Ethiopia that admitted students from twelve states in the region, representing a variety of sociocultural backgrounds. While Arba Minch University’s study area is limited, its diverse student body comes from all over the nation. For the 2023–2024 academic year, Arba Minch University reported having 1922 regular students enrolled in its BA program across four colleges, two institutes, and two schools. From this total, 1922 potential students were selected using Yamane’s formula [40]. The formula *n* = N/1 + N (e^2) was used, where n represents the sample size, e^2 represents the precision level (0.05%), and 1 represents the probability of the event occurring. This formula was chosen due to the lack of prior research as a reference point for the study, and it is preferred for applications with a 5% error margin and a 95% confidence level. Additionally, according to Gebremariam [41], if a research question focuses on the size of a parameter and a researcher collects enough data to produce an estimate with the desired level of accuracy, the sample should be chosen to provide a comprehensive conclusion to the research question.

The information for this study was provided by the university’s registrar and alumni manager. The study focused on students randomly selected from the Social Science and Humanities stream, despite the university’s goal of providing equal opportunities for enrollment across all colleges. Each participant was an adolescent following the standard curriculum. Using a purposiv sampling technique, 300 participants—224 male and 76 female students—were carefully selected as it is the probability sampling technique which depends upon the availability of the population (Refer to Table 1).

### Data collection tool

The researchers utilized a self-reported questionnaire to assess the psychosocial issues and their effects on the academic performance of the participants in the study. The questionnaire comprised two sections: a demographic questionnaire pertinent to the study’s objectives, and a measurement tool. The measurement tool consisted of 17 questions previously used in a study of Ugandan university students by Nsereko et al. [31]. These questions were integrated with the demographic questionnaire to create a single questionnaire containing multiple-choice questions. Participants were instructed to consider their psychosocial problems related to antisocial behavior (defined as actions that violate the rights of others or cause harm, such as theft, physical assault, and harassment, as well as non-criminal behaviors like lying and manipulation), emotions (typically associated with mental health conditions like mood disorders, relationship difficulties, or financial strain), trauma (an intense response to another person’s experiences, characterized by negative effects, behaviors, and emotions due to secondary exposure to traumatic events), and academics (encompassing a variety of issues like learning difficulties, procrastination, poor study habits, confusion or disinterest in learning, test anxiety, and teacher neglect or lack of attention) while answering the questions. Participants were asked to indicate their agreement level with the statements by circling the points ranging from 4 (Always) to 0 (Never at all) next to the provided response options. There were no correct or incorrect answers, and participants were encouraged to respond promptly. While there is no previous model for the reliability reference regarding this study, a pilot study was conducted on 23 students two weeks before the main data collection. The purpose of this pilot study was to check the reliability and validity of the instrument, with input from two counseling psychology experts. Although the questionnaire had not been validated in Ethiopia previously, the Cronbach’s alpha coefficients for each psychosocial problem were calculated for both the USEPP total scale (0.81) and the four subscales: emotional problems (0.70), antisocial behavior (0.73), traumatic experiences (0.60), and academic problems (0.63). The reliabilities for all domains and the total USEPP were considered satisfactory based on Kirk’s guidelines [42], which establish a conventional cutoff criterion for an acceptable alpha statistic of the scale.

### Procedures

The participants in this study were invited to participate by requesting permission to take part in the study. They completed a self-reported psychosocial problems identification questionnaire during class time. To assess the reliability and validity of the measurements, the questionnaire utilized a five-point Likert Scale with the following response options: Strongly Always (4), Often (3), Sometimes (2), Rarely (1), and Never at all (0). The questions for each psychosocial problem were presented as multiple-choice questions to minimize bias and discourage students from selecting the undecided, not sure, or don’t know options due to a lack of understanding or boredom/inattention, as recommended [43]. Before completing the questionnaires, students received an explanation of the purpose and instructions on how to fill them out. Their names were kept confidential to ensure privacy. In the questionnaire introduction, students were instructed to assign themselves a unique number from 1 to 300 to avoid interference with the psychosocial problems.

### Data analysis techniques

The data collected from Arba Minch University students through a self-report questionnaire was analyzed using SPSS 23 (Statistical Package for Social Science, Chicago, Illinois). After coding and checking the data quality, descriptive statistics were used to analyze the percentiles and frequencies, including Exploratory Factor Analysis (EFA) and Confirmatory Factor Analysis (CFA) in Amos 23. The results of the EFA indicated that the solution was based on the expected four factors of psychosocial problems, with all items loading on their respective factors. The factor solution explained 88.1% of the total variance, demonstrating good validity. Furthermore, CFA validation using Amos 23 was conducted to assess the reliability, convergent validity, and discriminant validity factors of the study data.

In addition, logistic regression analysis was used to investigate the association between the factors and psychosocial problems (emotional problems, antisocial behavior, trauma experiences, and academic problems). Bivariate logistic regression was employed to identify variables and estimate the probability of experiencing psychosocial problems based on the associated factors. Variables that showed significance in the bivariate logistic regression analysis were included in the multivariate logistic regression analysis. The Hosmer-Lemeshow test (*p* > 0.05) and the Variance Inflation Factor (VIF) (< 10) test were used to assess multicollinearity and the model’s goodness of fit, respectively. Odds ratios (OR) and 95% Confidence Intervals (CI) were used to determine the components related to the outcome variable. Statistical significance was considered at a *p*-value < 0.05.

## Results

To achieve the objectives of the study, quantitative data were used. The questionnaire was administered to university students. The quantitative data from the questionnaire was imported into SPSS 23 and analyzed in two steps; preliminary and main statistical process.

### Preliminary data results

The first step was to quantitatively explore the psychosocial problems of the students, and the second step was to investigate the relationship between these problems and the academic performance of the students.

**Table 1:** Socio-demographic information of respondents

		Frequency	Percent	Valid Percent	Cumulative Percent
Gender	Male	224	74.7	74.7	74.7
	Female	76	25.3	25.3	100.0
Religion affiliation	Orthodox	161	53.7	53.7	53.7
	Muslim	42	14.0	14.0	67.7
	Protistant	91	30.3	30.3	98.0
	Other	6	2.0	2.0	100.0
Marital status	Single	281	93.7	93.7	93.7
	Married	13	4.3	4.3	98.0
	Other	6	2.0	2.0	100.0
University Residence Location	On-campus	293	97.7	97.7	97.7
	Off-campus	7	2.3	2.3	100.0
Years in the university	First year	279	93.0	93.0	93.0
	Second year	14	4.7	4.7	97.7
	Fourth year	7	2.3	2.3	100.0
Living parents during holidays	Yes	195	65.0	65.2	65.2
	No	104	34.7	34.8	100.0
Education Sponsorship	Parents	258	86.0	86.3	86.3
	Relatives	9	3.0	3.0	89.3
	Governement	28	9.3	9.4	98.7
	Other	4	1.3	1.3	100.0
Age	Below 20	47	15.7	15.7	15.7
	20–24	246	82.0	82.0	97.7
	Above 24	7	2.3	2.3	100.0

Table 1 provides the socio-demographic information of the respondents. In terms of gender, 74.7% were male and 25.3% were female. Regarding religious affiliation, 53.7% identified as Orthodox, 14% as Muslim, 30.3% as Protestant, and 2% as other. In terms of marital status, 93.7% were single, 4.3% were married, and 2% were other. The majority of respondents (97.7%) lived on campus, while 2.3% lived off campus. In terms of year level at the university, 93% were first year students, 4.7% were second year students, and 2.3% were fourth year students. When asked if they lived with their parents during holidays, 65% answered yes and 34.7% answered no. In terms of education sponsorship, 86% were sponsored by their parents, 3% by relatives, 9.3% by the government, and 1.3% by other sources. The age range of the respondents was as follows: 15.7% were below 20 years age, 82% were between 20 and 24 years age, and 2.3% were in above 24 age ranges.

Before analyzing the quantitative data using correlational statistics, an Exploratory Factor Analysis (EFA) was conducted. The maximum likelihood method with varimax rotation was used to analyze the factor structure and correlations between items included in the scale. The construct validity of the data was assessed using Kaiser-Meyer-Olkin measures and Bartlett’s test of sphericity (Df (136), X2 = 1394.730, *p* = 0.001). The results of the EFA conducted in SPSS indicated that the Kaiser-Meyer-Olkin measure was 0.842, which is above the recommended threshold of 0.80. This suggests that the sample from which the data were collected was adequate. Additionally, the Bartlett’s test of sphericity was statistically significant at X2/df(136) = 1394.730; *p* = 0.001), indicating that the data were suitable for factor analysis. To ensure the accuracy of item grouping within each construct, the researcher performed an exploratory factor analysis. This analysis helped identify independent factors and determine which items loaded onto these factors (See Table 2).

**Table 2:** Reliability and convergent validity (*n* = 300)

IC	Items	EC	RCM	Mean	SD
EP1	Feeling stressed, being in low mood	0.730	0.850	2.18	1.20
EP2	Involved in one way or the other in academic mal practice	0.987	0.712	1.71	1.28
EP3	Often lacking welfare/pocket money for personal use	0.958	0.557	1.55	1.18
EP4	Low academic grades	0.967	0.899	1.19	0.92
EP5	I sometimes find it difficult to sleep or I sleep too much	0.940	0.653	1.70	1.27
ASB1	Sometimes experiencing wishes of being dead	0.876	0.847	1.07	1.26
ASB2	Adjustment problems in my new environment, i.e., hostel, campus life	0.978	0.697	1.74	1.21
ASB3	Inadequate study skills to meet university academic demands	0.836	0.456	1.31	1.15
ASB4	Uncontrolled drinking of alcohol	0.858	0.897	0.59	1.02
ASB5	Gambling/betting for financial gain	0.750	0.901	0.80	1.28
TE1	I have problems of concentrating in life generally	0.964	0.904	1.35	1.21
TE2	I am not able to concentrate on my studies as I would have liked	0.895	0.926	1.60	1.25
TE3	I take sexual advantage of others	0.923	0.918	0.52	0.90
TE4	My family is experiencing problems of which I am of great concern	0.895	0.933	1.19	1.33
AP1	Experiences of irrational fears/phobia	0.754	0.950	1.39	1.32
AP2	Unpredictable/Insecure tuition fees status	0.814	0.931	1.39	1.24
AP3	I am involved in behaviours I should be ashamed of if they became public	0.857	0.864	1.19	1.29

Note: IC = item code; EC = extraction communality; RCM = related component matrix; M = mean; SD = standard deviation

**Fig. 2:**
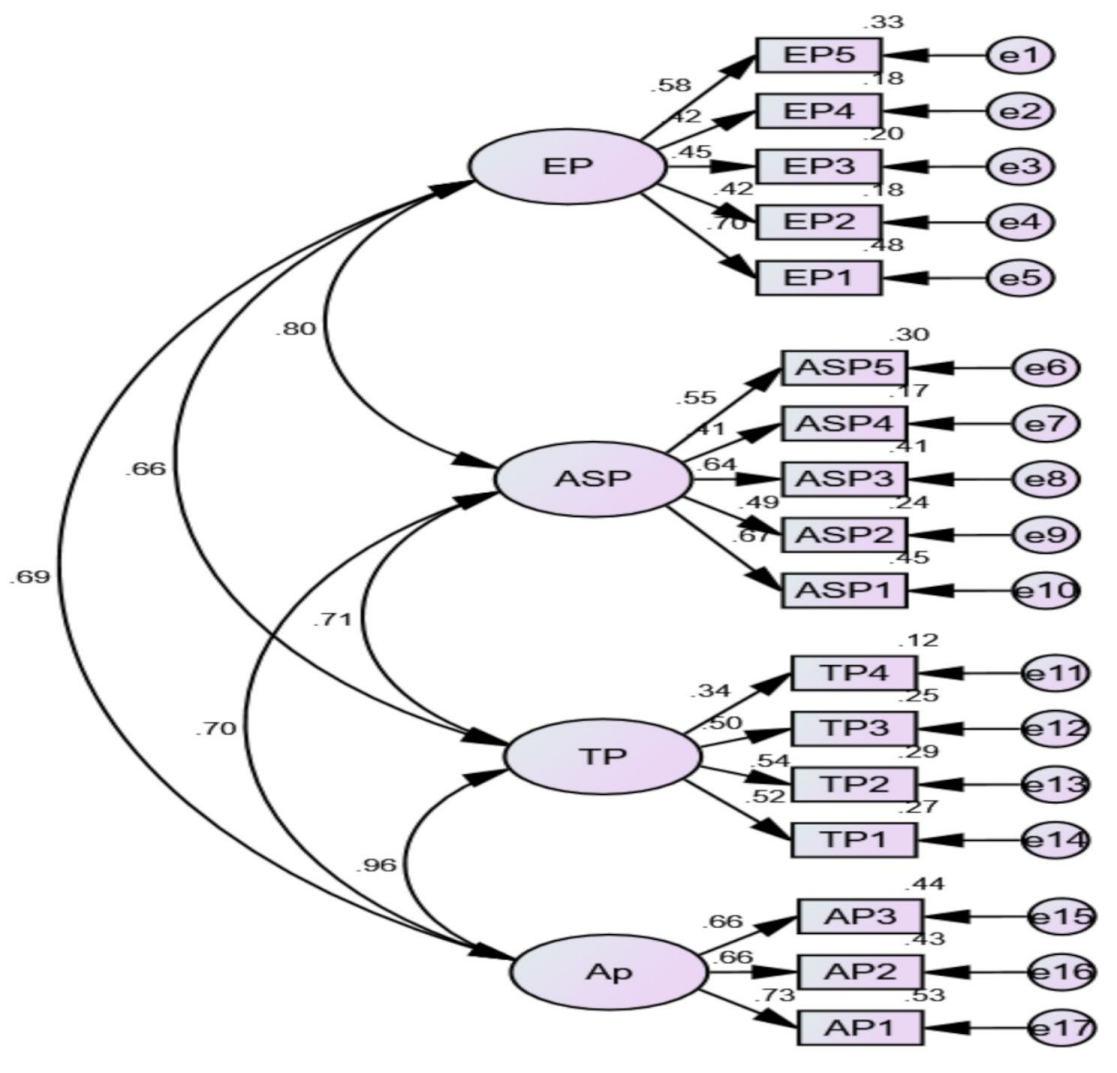
Conffirmatory factor analysis Note: EP = emotional problems; ASP = antisocial problems; TP = trauma problems; AP = academic problems (for complete informatics refer to data collection instruments section and Table 3)

According to the results in Table 2, the extraction communality ranged from 0.730 to 0.987, while the factor loadings ranged from 0.557 to 0.950. All standardized factor loadings for the items were above 0.50, indicating good convergent validity. The factor analysis confirmed that the data collection instruments were suitable for further analysis in this study. Additionally, the research analysis utilized information gathered from the questionnaire provided to the respondents. To assess and determine the participants’ experiences with reflective practice, the Pearson correlation coefficient was employed (Fig. 2 presents the questionnaire items by CFA).

Figure 2 shows that the results of the CFA indicate that the model had good fit statistics, including X2/df = 4.008, RMSEA of 0.131, RMR of 0.076, and GFI of 0.745. The recommended values, based on the guidelines of Browne and Cudeck (1992), are provided in the brackets (RMSEA < 0.05, RMR < 0.05, GFI > 0.90). All items’ standardized factors had loadings above 0.50, indicating good convergent validity. Another piece of evidence of convergent validity is that the maximum shared variance is less than the respective average variances extracted for all variables. The Cronbach’s alpha and composite reliability for all variables are above 0.80, demonstrating good reliability.

### Prevalence of psychosocial problems

After the preliminary data analysis, the first research question of this study was examined. This question focuses on the prevalence of psychosocial problems, such as emotional issues, antisocial behavior, trauma experiences, and academic problems, among Arba Minch University students. The results of this analysis can be found in Table 3.

**Table 3:** The prevalence of psychosocial problems among university students

Variables	Measures/Levels	Frequency	Percent
Emotional problems	High/Severe	20	6.7
	Moderate	139	46.3
	No/Low	141	47.0
Antisocial behavior	High/Severe	15	5.0
	Moderate	164	54.7
	No/Low	121	40.3
Trauma experiences	High/Severe	21	7.0
	Moderate	71	23.7
	No/Low	208	69.3
Academic problems	High/Severe	25	8.3
	Moderate	69	23.0
	No/Low	206	68.7

The results showed that only 6.7% of the total respondents had severe/high emotional psychosocial problems, while 46.3% had moderate emotional problems, and 47% had no/low emotional problems. In terms of antisocial problems, 5% of respondents were severe/high, 54.7% were moderate, and 40.3% were no/low. Regarding trauma problems, 7% of higher education students reported high/severe issues, 23.7% reported moderate problems, and 69.3% reported no/low problems. Lastly, in relation to academic problems, 8.3% of students reported high/severe issues, 23.7% reported moderate issues, and 68.3% reported no/low issues (See Table 3).

### Association of socio-demographic factors with psychosocial problems

Furthermore, in order to investigate the factors associated with psychosocial problems, descriptive (crosstabs) and inferential (bivariate and multivariate logistic regression analysis) were performed. The purpose was to identify the factors associated with the multivariate analysis. The results were then analyzed to determine the association between the socio-demographic information of the respondents and their emotional problems, antisocial behavior, trauma experiences, and academic problems, which are types of psychosocial problems. Additionally, after checking the assumptions of binary logistic regression, variables in the bivariate analysis that were statistically associated with psychosocial problems (with a *p*-value of less than 0.25) were exported to the multivariate logistic regression to identify the associated factors. Gender, religion affiliation, marital status, living situation during holidays, and educational sponsorship were shown to be statistically significant in one or more dependent variables. (Specifically, refer to Tables 4, 5 and 6, and Table 7).

**Table 4:** Factors associated with emotional problems using bivariate analysis

Associated factors / independent		Category		Bivariate Analysis	Multivariate Analysis
		No	Yes	OR (95% CI)	OR (95% CI)
Gender	Male	163	61	0.456 (0.204, 1.019)*	0.508 (0.243, 1.060)
	Female	65	11		
Religious affiliation	Orthodox	116	45	0.493 (0.194, 0.251)*	2.930 (0.551, 15.583)
	Muslim	35	7	0.371 (0.159, 0.868)*	6.460 (1.033, 40.405)**
	Protestant	74	17	0.000 (0.000, ---)**	5.825 (1.036, 32.769)**
	Other	3	3		
Marital Status	Single	219	62	1.842 (0.326, 10.405)	4.180 (0.786, 22.231)
	Married	6	7	3.534 (0.547, 22.834)*	2.266 (0.278, 18.497)
	Other	3	3		
University residence	On-compass	224	69	2.683 (0.470, 15.314)	
	Off-compass	6	3		
Years in the university	First year	214	65	1.754 (0.317, 9.712)	
	Second year	3	14	3.854 (0.329, 45.136)	
	Fourth year	4	7		
Living home during holidays	Yes	143	52	0.432 (0.207, 0.901)**	0.477 (0.247, 0.921)**
	No	85	19		
Education Sponsorship	Parents	202	56	9.188 (1.679, 50.279)**	4.010 (0.518, 31.042)
	Relatives	4	5	1.781 (0.666, 4.761)*	0.624 (0.052, 7.423)
	Gov’t	20	8	0.942 (0.040, 22.417)	2.330 (0.270, 20.076)
	Other	2	2		
Age	Below 20	40	7	1.829 (0.718, 4.655)*	19.193 (1.802, 204.370)**
	20–24	187	59		13.021 (1.416, 119.761)**
	Above 24	1	6		

Notes: Variable(s) entered on step 1: Gender, Religion, Marital status, University residence, Year level, Living home during holydays, Education sponsor, Age. * less than 0.25 *p*-values selected for multivariate analysis; **significant at 0.05 *p*-values

Table 4 displayed the association between socio-demographic information and types of emotional problems among adolescent students at Arba Minch University. The results indicated that only two socio-demographic factors, religion affiliation, and educational sponsorship, were significantly associated with emotional problems and predicted moderate or high/severe problems among respondents after controlling for other factors. Specifically, Protestant respondents were more likely to have moderate and high/severe emotional problems (OR = 0.000, 95% CI = 0.000**). Respondents who reported living at home during holidays were also more likely to have moderate and high/severe emotional problems (OR = 0.432, 95% CI = 0.207, 0.901**). In contrast, respondents with parents as educational sponsors were more likely to have moderate and high/severe emotional problems (OR = 9.188, 95% CI = 1.679, 50.279**). Other socio-demographic factors did not show significance at *p* < 0.05. The significance levels for respondents’ socio-demographic information, except for Protestant religion, living at home during holidays, and parental educational sponsorship, ranged from 0.056 to 0.971.

Furthermore, multivariate analysis revealed that religion affiliation, living at home during holidays, and educational sponsorship significantly predicted moderate to extremely high/severe symptoms among respondents after controlling for other factors. Specifically, Muslims (OR = 6.460, 95% CI = 1.033, 40.405**) and Protestants (OR = 5.825, 95% CI = 1.036, 32.769**) were more likely to have moderate and high/severe emotional problems based on religion affiliation. Respondents who reported living at home during holidays were less likely to have moderate and high/severe emotional problems (OR = 0.477, 95% CI = 0.247, 0.921**).

**Table 5:** Factors associated with antisocial behavior using bivariate analysis

Associated factors / independent		Category		Bivariate Analysis	Multivariate Analysis	
		No	Yes	OR (95% CI)	OR (95% CI)	
Gender	Male	179	53	0.644 (0.321, 1.212)		
	Female	59	17			
Religious affiliation	Orthodox	114	47	0.065 (0.169, 1.056)	3.825 (0.678, 21.571)*	
	Muslim	33	9	0.001 (0.076, 0.522)**	7.333 (1.153, 46.662)**	
	Protestant	80	10	0.999 (0.000, ---)	12.346 (2.028, 75.142)**	
	Other	2	4			
Marital Status	Single	220	1	0.000 (0.000, ---)		
	Married	6	-	2.180 (0.357, 13.326)		
	Other	3	-			
University residence	On-compass	223	1	0.509 (0.059, 6.748)		
	Off-compass	6	-			
Years in the university	First year	218	60	7.894 (7.894, 31.744)**	4.781 (0.897, 25.485)*	
	Second year	8	6	4.394 (4.394, 51.535)*	0.983 (0.128, 7.573)	
	Fourth year	3	3			
Living home during holidays	Yes	148	46	0.803 (0.407, 1.585)		
	No	80	24			
Education Sponsorship	Parents	208	1	25.572 (0.000, ---)	11.814 (1.199, 116.429)**	
	Relatives	-	-	2.128 (0.855, 5.301)*	2.011E-7 (0.000, ---)	
	Gov’t	19	-	1.772 (0.063, 50.089)	4.896 (0.449, 53.354)	
	Other	2	-			
Age	Below 20	35	12	0.567 (0.262, 1.227)*	91.410 (11.476, 23.206)**	
	20–24	194	51	0.803 (0.000, ---)	51.270 (51.270, 51.870)	
	Above 24	-	7			

Notes: Variable(s) entered on step 1: Gender, Religion, Marital status, University residence, Year level, Living home during holydays, Education sponsor, Age. * less than 0.25 *p*-values selected for multivariate analysis; **significant at 0.05 *p*-values

Table 5 shows the bivariate analysis between antisocial psychosocial problems and the socio-demographic factors of the respondents. The results indicate that while most socio-demographic factors are not statistically significant at *p* < 0.05, religion affiliation and year levels were significantly associated with moderate or high/severe problems among the targeted students. Specifically, being Muslim (OR = 0.001, 95% CI = 0.076, 0.522**) and in the first year (OR = 7.894, 95% CI = 7.894, 31.744**) were more likely to have moderate or high/severe antisocial problems. Conversely, there was no significant association between other socio-demographic factors and antisocial problems, with levels ranging from 0.065 to 0.999.

Furthermore, the multivariate analysis revealed that religion affiliation was a significant predictor of moderate or high/severe problems among the respondents at Arba Minch University. Specifically, being Muslim (OR = 7.333, 95% CI = 1.153, 46.662**) and Protestant (OR = 12.346, 95% CI = 2.028, 75.142**), having parent sponsorship for education (OR = 11.814, 95% CI = 1.199, 116.429**), and being single (OR = 91.410, 95% CI = 11.476, 23.206**) were more likely to have moderate or high/severe antisocial problems after controlling for other factors.

**Table 6:** Factors associated with trauma experiences using bivariate analysis

Associated factors / independent		Category		Bivariate Analysis	Multivariate Analysis	
		No	Yes	OR (95% CI)	OR (95% CI)	
Gender	Male	177	47	0.264 (0.095, 0.736)**	0.939 (0.121, 7.309)	
	Female	71	5			
Religious affiliation	Orthodox	131	30	0.097 (0.013, 0.743)**	9.968 (0.590, 168.309)	
	Muslim	41	1	0,685 (0.289, 1.621)	1.164 (0.160, 8.461)	
	Protestant	73	18	0.548 (0.025, 12.154)	0.293 (0.110, 0.781)**	
	Other	3	3			
Marital Status	Single	237	44	40.501 (3.722, 440.692)**	1.498E-8 (3.76E, 5.95E)**	
	Married	5	8	0.000 (0.000, ---)	2.37E-9 (2.37E-9, 2.37E-9)	
	Other	6	-			
University residence	On-compass	224	49	3.970 (0.680, 23.172)*	3.783 (0.819, 17.485)	
	Off-compass	4	3			
Years in the university	First year	231	49	2.950 (0.615,14.138)*	0.841 (0.099, 7.153)	
	Second year	11	3	1.839 (0.154, 21.997)	0.611 (0.052, 7.240)	
	Fourth year	6	1			
Living home during holidays	Yes	162	33	1.171 (0.554, 2.476)		
	No	85	19			
Education Sponsorship	Parents	212	46	0.000 (0.000, ---)		
	Relatives	9	-	1.233 (0.397, 3.833)		
	Gov’t	22	6	0.000 (0.000, ---)		
	Other	2	-			
Age	Below 20	39	8	0.838 (0.318, 2.212)		
	20–24	205	41	0.289 (0.012, 6.958)		
	Above 24	4	3			

Notes: Variable(s) entered on step 1: Gender, Religion, Marital status, University residence, Year level, Living home during holydays, Education sponsor, Age. * less than 0.25 *p*-values selected for multivariate analysis; **significant at 0.05 *p*-values

Table 6 presents the bivariate analysis between the type of trauma and psychosocial experiences, and the association with the socio-demographic factors of respondents. The results showed that gender, religion affiliation, and marital status significantly influenced the likelihood of having moderate or severe trauma problems among the targeted students. Specifically, being male (OR = 0.264, 95% CI = 0.095, 0.736**), having an orthodox religion affiliation (OR = 0.097, 95% CI = 0.013, 0.743**), and being single (OR = 40.501, 95% CI = 3.722, 440.692**) were statistically significant factors.

Other socio-demographic factors of the respondents were not significantly associated with trauma-based psychosocial problems (*p* > 0.05), with significance levels ranging from 0.126 to 0.999 alphas. Furthermore, the multivariate analysis indicated that religion affiliation and marital status were significant predictors of moderate or severe trauma problems among Arba Minch University students. Specifically, those with a protestant religion affiliation (OR = 0.293, 95% CI = 0.110, 0.781**) were more likely to experience moderate or severe trauma problems. Additionally, single individuals (OR = 1.498E-8, 95% CI = 3.767E-9, 5.956E-8**) were more likely to experience moderate or severe trauma problems after controlling for other associated factors.

**Table 7:** Factors associated with academic problems using bivariate analysis

Associated factors / independent		Category		Bivariate Analysis	Multivariate Analysis	
		No	Yes	OR (95% CI)	OR (95% CI)	
Gender	Male	169	55	0.452 (0.199, 1.028)*	0.433 (0.201, 0.932)**	
	Female	67	9			
Religious affiliation	Orthodox	125	36	1.075 (0.452, 2.556)		
	Muslim	33	9	0.866 (0.390, 1.923)		
	Protestant	72	19	0.000 (0.000, ---)		
	Other	6	-			
Marital Status	Single	221	60	2.387 (0.490, 11.618)		
	Married	9	4	0.000 (0.000, ---)		
	Other	6	-			
University residence	On-compass	214	61	2.375 (0.454, 12.414)		
	Off-compass	4	4			
Years in the university	First year	222	57	2.482 (0.535, 11.579)*	4.373 (0.947, 20.205)	
	Second year	11	3	38.244 (0.000, ---)	3.587 (0.489, 26.310)	
	Fourth year	3	4			
Living home during holidays	Yes	157	44	0.685 (0.338, 1.388)		
	No	85	19			
Education Sponsorship	Parents	204	54	1.818 (0.332, 9.961)		
	Relatives	6	3	1.019 (0.334, 3.110)		
	Gov’t	23	5	0.000 (0.000, ---)		
	Other	3	1			
Age	Below 20	36	11	0.729 (0.320, 1.663)		
	20–24	196	50	52.674 (0.000, ---)		
	Above 24	4	3			

Notes: Variable(s) entered on step 1: Gender, Religion, Marital status, University residence, Year level, Living home during holydays, Education sponsor, Age. *less than 0.25 *p*-values selected for multivariate analysis; **significant at 0.05 *p*-values

Table 7 presents the bivariate analysis between the problems of academic type of psychosocial variables. The results of a study on factors associated with academic type of psychosocial problems were not statistically significant at the 0.05 alpha level. More specifically, the significance levels ranged from 0.058 to 0.999. On the other hand, the multivariate analysis of the targeted respondents showed that the gender variable was the only significant predictor of having moderate or high/severe academic problems (OR = 0.433, 95% CI = 0.201, 0.932**) among Arba Minch University students after controlling for other associated factors.

## Discussion

The present study focused on psychosocial problems [31], including emotional problems, antisocial behavior, trauma experiences, and academic problems, with reference to the socio-demographic information of adolescent students at Arba Minch University, Ethiopia. The findings of this study regarding the prevalence of psychosocial problems among adolescent students at Arba Minch University revealed that 5–8.3% of the respondents had high/severe psychosocial problems, while 23–54.7% had moderate psychosocial problems. On average, nearly half of the respondents had high/severe and moderate psychosocial problems (emotional problems, antisocial behavior, trauma experiences, and academic problems). More specifically, the respondents encountered emotional problems (6.7% high/severe and 46.3% moderate), antisocial behavior (5% high/severe and 54.7% moderate), trauma experiences (7% high/severe and 23% moderate), and academic problems (8.3% high/severe and 23% moderate). The prevalence of no/low psychosocial problems was 47%, 40.3%, 69.3%, and 68.7%, respectively. These findings are consistent with a previous study by Sharma et al. [24] conducted in Dehradun, which confirmed that 40.5% of individuals had such psychosocial problems.

Similarly, according to Abera et al. [6], the prevalence of psychosocial problems in terms of depression, anxiety, and stress was 52.1%, 52.6%, and 22.6% among high school students, respectively. The prevalence of the current study is somewhat related to a study by Bista et al. [25] that used a cross-sectional design to identify the prevalence of psychosocial problems among Nepalese students, ranging from 17.03 to 31.2%. Additionally, the prevalence of psychosocial problems among adolescents was 32.4%. The study by Latiff et al. [18] concluded that the prevalence of psychosocial issues was 45.6%, with factors associated with depression at 46%, anxiety at 59.1%, and stress at 38.1%. These results indicated that psychosocial problems in adolescents were influenced by socio-demographic factors. For example, gender and age were significant predictors, while in the current study; gender roles did not influence psychosocial problems. However, the findings of the current study are higher than those of a study conducted by Timalsina et al. [3], which found that 12.9% of teenagers in Nepal experienced psychosocial problems, and the prevalence of psychosocial problems was 20.7% [12]. On the other hand, the findings are lower than those of a study conducted by Latiff et al. [18], which studied the prevalence of psychosocial problems in Malaysia and revealed that the prevalence of psychosocial problems, including externalizing (24.9%) and internalizing (20.7%), was high/severe when compared to the moderate psychosocial problems experienced by respondents. The discrepancies might be due to differences in sample size, measurement scale, cut-off points for psychosocial problems, and the types of well-researched psychosocial problems. Some studies included a large number of participants (e.g., Abera et al. [6], included *n* = 654), and some studies used different measurement tools (e.g., Epidemiological Studies-Depression Scale; Patient Health Questionnaire).

The findings regarding the associated factors with psychosocial problems indicate that each of the updated psychosocial problems (emotional, antisocial, trauma, and academic) have differences. Particularly, the prevalence of emotional problems is 6.7% high/severe and 46.3% moderate. Statistically significant associated factors include religion affiliation (Muslim and Protestant), living at home during holidays, and age group below 20 years as predictors for emotional problems among the respondents. This finding is in line with a previous study [18] which reported a significant association between parents’ relationship, religion affiliation, age groups, and psychosocial problems. On the other hand, the finding of the present study contradicts Shrestha et al. [44], which revealed no association with emotional problems. Therefore, the finding of emotional psychosocial problems advances the literature of previous studies [9, 29, 45] conducted on psychosocial and health problems in different ecological environments, revealing that the transition from upper secondary school to higher education institutions is a major life change for many adolescents.

Regarding antisocial behavior, 5% of the respondents had high/severe and 54.7% had moderate levels. This finding reveals that most respondents in this study experienced at least moderate emotional psychosocial problems. Significantly associated factors with antisocial behavior were religion affiliation (Muslim and Protestant), educational sponsorship by parents, and age group below 20 years as predictors for antisocial behavior among respondents. While Latiff et al. [18] revealed that age group and family sponsorship significantly predicted moderate to extremely severe antisocial psychosocial problems, perceived antisocial behavior has been shown to predict low levels of adjustment to university programs and activities. Other previous studies [1, 8] revealed self-esteem, social support, and emotional intelligence as predictors of better adjustment. However, the antisocial behavior focused finding contradicts the literature of Hailemariam et al. [14] which indicates that attending higher education offers students learning experiences and opportunities for psychosocial development, while Garcia et al. [26] suggest that entering higher education may be a source of strain and acute stress.

Furthermore, 7% of the respondents in the current study had high/severe and 23% had moderate trauma experiences. According to Beharu [33], this is a high and dangerous interpretation. Additionally, the statistically significant associated factor with trauma experiences is marital status; single respondents experienced more trauma than married respondents and other demographic factors in the present study. This finding is supported by the study conducted by Shrestha et al. [44] which indicates the Shrestha et al. [44] study shows that the prevalence of psychosocial problems among adolescents was 32.4%. Similarly, Tvedt & Bru [36] demonstrated that being single adolescents increases the risk of psychosocial problems. However, gender was not significantly associated with any of the psychosocial problems addressed in the study, except for male students in academic problems. The academic type of psychosocial problems was related to the factors associated in this study.

The factors associated with academic problems indicate that male respondents are significantly more likely to experience psychosocial issues than female respondents and other demographic groups. This finding aligns with existing literature which suggests that the demands of higher education and new social relationships can lead to psychosocial challenges among students [21]. Similarly, Ozturk [12] concluded most of the adolescents were internet users and one in five adolescents was at risk of psychosocial problems. Internet addiction and psychosocial problems were associate with several socio-demographic factors. As students transition to higher education, they often experience reduced contact and social support from friends and family, which can contribute to emotional difficulties [20, 25]. Given the negative impact of these stressors on Ethiopian students pursuing higher education, it is crucial to take further steps to address these challenges.

Overall, the study revealed a high prevalence of psychosocial problems among Ethiopian university students. The findings showed that emotional, antisocial, trauma, and academic issues were significantly associated with the socio-demographic characteristics of the students at a significance level of *p* < 0.05. This suggests that psychosocial problems have a significant impact on the well-being of adolescent students. While the revious research [44, 45] has shown that students generally have positive attitudes towards their academics, despite some academic performance limitations [7, 24], this study implies the need to update and revise the psychosocial health of university students, which is crucial for improving psychosocial problems. This can be achieved through increasing the allocation of healthcare budget for awareness programs and providing proper knowledge in targeted universities, as well as in all higher institutions in the country, based on the findings of this study. Additionally, high school students have been found to have positive emotions related to academic performance. On the other hand, N-yelbi et al. [2] have highlighted the distinction between psychosocial and academic problems, suggesting that students may need specific support to manage their emotions during university studies. In light of these previous studies, it is evident that psychosocial problems are a significant concern among university students in Ethiopia.

## Limitations for future studies

While the present study extends previous research on psychosocial problems affecting university students in several important directions, it has some limitations that should be addressed in future research. Firstly, the study focused only on the Ethiopian context, particularly on adolescent students at Arba Minch University. This focus may not capture the full range of psychosocial problems experienced by adolescent students the Ethiopian universities, especially those highlighted in many previous studies: depression, anxiety, and stress. Secondly, the representativeness of the sample and the generalizability of the findings are two further constraints that should be acknowledged and taken into account. Primarily, self-selection sample bias may be present because the data were collected through a self-reported questionnaire. Additionally, since the participants volunteered, a professional bias may have influenced the sample, leading to a lack of balance in representing the study’s ecological contexts. Furthermore, the sample used in this study may not be representative, and the results may not be generalizable to all university adolescent students, especially those outside of Ethiopia. Lastly, the location of the study may have also influenced the findings. Specifically, the ecological site of the study may have influenced the findings since the study exclusively focused on Ethiopian university students, potentially limiting a comprehensive understanding of the psychosocial problems. Therefore, some recommendations for future studies are made in light of these limitations.

To gain a more validated and comprehensive understanding of the psychosocial problems and associated factors among higher education adolescent students, future research endeavors should include a wider range of participants, including those from private and public universities and colleges. It would be beneficial for future scholars to explore university students in more detail, not limited to those at Arba Minch University in Ethiopia. The results of this study could also be relevant to other university-based studies, including those in developing countries grappling with psychosocial problems and associated factors. Therefore, the present study suggests that psychosocial problems and associated factors should be considered by university students for their self-efficacy and resilience in self-identification, as well as by scholars who apply interventions on adolescents’ psychosocial problems.

## Conclusion

The findings of the present study indicate a prevalence of psychosocial problems ranging from 5 to 8.3% in high/severe cases, and from 23 to 54.3% in moderate cases. The factors associated with each type of psychosocial problem are also identified. Religion affiliation, living situation during holidays, and age groups are significantly linked to emotional problems. When it occurs to antisocial behavior, religion affiliation, educational sponsorship, and age group were significant predictors of respondents’ psychosocial well-being. For other types of psychosocial problems, factors such as being single in marital status were more prevalent in trauma experiences, and gender group was significantly associated with academic-related psychosocial issues. These findings underscore the importance of implementing effective psychosocial self-efficacy training in Ethiopian public universities. It is recommended that psychosocial support be integrated into the curriculum to enhance overall student experience. This study has implications for the field of education, highlighting the importance of addressing psychosocial problems in schools. The role of psychosocial issues in educational settings, particularly in university academic instruction, both inside and outside the classroom, is emphasized. Additionally, addressing the gap in research on updated psychosocial problems such as emotional issues, antisocial behavior, trauma, and academic difficulties is essential for improving the mental health and academic success of higher education students in Ethiopia. By expanding the focus of studies to include these areas, educators, policymakers, and mental health professionals can develop more effective strategies to support students and enhance their future prospects.

## Data Availability

All data are available upon request from the editor, and the corresponding author can provide them.

## Abbreviations

AP: Academic Problems
ASB: Antisocial Behavior
CFA: Confirmatory Factor Analysis
DGC: Department Graduate Committee
EFA: Exploratory Factor Analysis
EP: Emotional Problems
TE: Trauma Expériences
